# Implementing UK Oncology Nursing Society–Informed Digital Symptom Triage With Episode-Based Review in Routine NHS Acute Oncology: Service Evaluation

**DOI:** 10.2196/92586

**Published:** 2026-05-21

**Authors:** Mohamed Sheeraz Mohamed Azhar, Rishabh Ray, Sagar Sharma, Hiten Chauhan, Margaret Ruth Robinson, Yetunde Owoseni, Anupama Vijay, Sadaqat Hussain

**Affiliations:** 1 University Hospitals of Leicester NHS Trust Leicester, England United Kingdom

**Keywords:** acute oncology, digital health, patient-reported outcomes, symptom monitoring, triage, UKONS, quality improvement, implementation science

## Abstract

**Background:**

Patients receiving systemic anticancer therapy (SACT) can deteriorate clinically between scheduled appointments; yet, acute oncology services often rely on reactive helplines with limited longitudinal symptom visibility.

**Objective:**

The aim of this study was to evaluate the feasibility, safety, and workflow integration of OncsCare, a digital symptom triage platform mapping patient-reported symptoms to UK Oncology Nursing Society (UKONS) acuity tiers with episode-based clinician review.

**Methods:**

This 10-week service evaluation (July to September 2025) implemented OncsCare within a UK tertiary acute oncology service. Patients completed daily symptom check-ins mapped to UKONS-informed green/amber/red tiers. Alerts were grouped into episode-level triage events using prespecified rules (48-hour window, symptom-domain continuity) to represent the operational workload. Outcomes included engagement, alert distribution, escalation pathways, timeliness of clinical response, and safety signals via structured case-finding.

**Results:**

Thirty-two patients participated (none withdrew) in this study. Daily check-in completion rate was 91.7% (1444/1574 expected patient-days). From 362 amber/red alerts, 62 episodes were generated; 38.7% (24/62) were clinically actionable, resulting in telephone management (n=12, 50%), acute care assessment (n=9, 37.5%), emergency referral (n=2, 8.3%), or admission (n=1, 4.2%). Median time to first clinical response for in-hours red alerts was 47 minutes. Predefined safety case-finding identified no intervention-attributable safety signals. Patients reported increased home reassurance (n=17, 85%), and clinicians reported improved situational awareness without increased workload.

**Conclusions:**

UKONS-informed digital triage with episode-based review demonstrated feasibility, with no intervention-attributable safety signals identified in this small single-center evaluation. This operational model addresses alert fragmentation and supports prospective multicenter evaluation.

## Introduction

Systemic anticancer therapy (SACT) is associated with acute toxicities that can evolve rapidly between scheduled clinical encounters. Symptoms such as diarrhea, vomiting, mucositis, dyspnea, and pain may precede time-critical complications, including infection, dehydration, and organ dysfunction. Acute oncology (AO) services therefore function as a safety net, aiming to identify deterioration early and deliver appropriate escalation through structured triage. In the United Kingdom, the United Kingdom Oncology Nursing Society (UKONS) triage tool provides a nationally adopted framework for grading symptom severity and specifying escalation timeframes [1].

Despite widespread use of UKONS triage during telephone contacts, many AO services remain predominantly reliant on reactive, patient-initiated helplines. This model can be delayed and variably accessible, depends on patients recognizing deterioration and initiating contact, and provides limited longitudinal visibility of symptom trajectories between contacts. A recent UK-wide review led by the UK Acute Oncology Society (UKAOS) identified marked variation in the organization, governance, digital maturity, and integration of SACT helplines, alongside inconsistent data capture and limited alignment with wider urgent and emergency care pathways [2].

Electronic patient-reported outcome (ePRO) systems have demonstrated that symptom monitoring with clinician response improves symptom control and survival [3,4]. More recent trials have extended this evidence: the PRO-TECT trial demonstrated improved patient-reported outcomes across metastatic cancer types using electronic monitoring [5], and the SYMPRO-Lung study showed improved 2-year survival for patients with lung cancer monitored with web-based symptom tools [6]. Phase III evidence from the eRAPID program has further established the value of ePRO integration in routine chemotherapy care [7]. However, many platforms are designed primarily for longitudinal tracking and research workflows rather than operational integration into acute care pathways, and few map patient-reported symptoms to nationally adopted triage frameworks.

To our knowledge, few implementations have operationalized patient-reported symptom monitoring within a nationally standardized AO triage framework. To address these gaps, we developed OncsCare, a clinician-designed digital symptom monitoring and safety-netting platform that maps daily patient-reported symptoms to UKONS-informed red/amber/green acuity tiers with episode-based grouping of temporally related alerts to reduce alert fragmentation. Evaluation end points were prospectively informed by the RE-AIM (Reach, Effectiveness, Adoption, Implementation, Maintenance) framework [8], which underpins the 5 objectives below; implementation determinants were mapped retrospectively using the Consolidated Framework for Implementation Research (CFIR) [9], as detailed in the Methods and Discussion. We conducted a 10-week service evaluation with five objectives: (1) engagement and retention, (2) alert distribution and episode-level actionability, (3) escalation pathways and timeliness of clinical response, (4) safety signals using structured case-finding, and (5) patient and clinician experience.

## Methods

### Study Design

We conducted a 10-week quality improvement service evaluation (July 11, 2025 to September 19, 2025) within the AO service at University Hospitals of Leicester NHS Trust, a UK tertiary cancer center serving approximately 1.1 million people. The intervention was implemented as an adjunct to existing AO pathways and did not replace standard care. Participants retained access to usual services, including the 24-hour AO helpline, Oncology–Hematology Assessment Unit (OHAU) pathway, and emergency department (ED) assessment.

Two complementary implementation science frameworks informed the evaluation design. RE-AIM [8] was applied prospectively to define evaluation end points and structure outcome selection across engagement, adoption, and implementation domains. The CFIR [9] was applied retrospectively to map determinants of adoption across 5 domains from qualitative clinician debrief data. Both frameworks are elaborated in the Discussion.

Prior to implementation, informal focus groups with 3 patients receiving SACT and 2 caregivers informed onboarding materials and safety messaging. Key themes included the importance of clear instructions to bypass the application and use urgent/emergency pathways when needed, transparency about monitoring processes, and minimizing the burden of daily check-ins. These insights were incorporated into onboarding scripts and in-application safety-netting content.

To contextualize the evaluation, AO helpline activity was described using digitized call logs from January 2023 to June 2024. Metrics included weekly call volume and call outcomes categorized as managed without admission, escalated to OHAU, referred to ED/admitted, or administrative/other. Baseline data were descriptive and not intended for formal statistical comparison with the pilot period.

### Participants

Eligible participants were adults (≥18 years) receiving active SACT with anticipated treatment duration ≥6 weeks, ECOG performance status 0-2, access to a smartphone or tablet (independently or with caregiver support), and ability to provide consent in English. Exclusion criteria included end-of-life care with estimated life expectancy <3 months, lack of capacity, enrolment in other intensive daily remote monitoring programs, or anticipated prolonged admission or travel during the pilot period. Patients were approached pragmatically during SACT visits, OHAU reviews, or outpatient appointments. Participants provided informed verbal consent documented in the clinical record using a standardized template.

### Setting and Intervention

OncsCare comprises a patient-facing mobile app (iOS/Android) and a secure clinician dashboard. Participants completed a brief daily symptom check-in covering temperature, dyspnea, nausea/vomiting, diarrhea, constipation, pain, bleeding/bruising, fatigue, skin rash/reaction, peripheral neuropathy, and oral mucositis. Symptom severity was captured using a 0-3 ordinal scale with patient-facing anchors co-designed with AO nurses, mapped to UKONS-informed acuity tiers. On submission, tier-specific end-screen guidance was displayed: green alerts provided reassurance, amber alerts advised monitoring while awaiting review, and red alerts instructed patients to contact the AO helpline urgently or attend ED if unwell. Red alerts generated real-time dashboard notifications; amber alerts were reviewed in scheduled twice-daily batches. Green submissions were available for the longitudinal context but did not require active clinician review. Dashboard monitoring occurred during weekday in-hours periods (Monday to Friday, 9 AM to 5 PM). This was distinct from the 24/7 AO helpline, which continued to operate across two 12-hour nursing shifts throughout the evaluation.

Alerts were grouped into episode-level triage events using a prespecified rule-based approach. An episode was initiated by any amber or red alert. Subsequent amber/red alerts were assigned to the same episode if they occurred within 48 hours of the most recent alert and shared symptom-domain continuity (gastrointestinal, respiratory, fever/temperature, pain, mucositis, neuropathy, fatigue/wellbeing, bleeding/bruising, skin). A new episode was created when the 48-hour window elapsed without qualifying alerts or when alerts reflected a distinct symptom pattern. Grouping consistency was reviewed periodically by the project lead.

Implementation followed Plan–Do–Study–Act (PDSA) cycles across 4 phases (Multimedia Appendix 1), iteratively refining onboarding processes, alert threshold calibration, dashboard efficiency (including episode-based alert grouping introduced in week 3), and safety-netting messaging in response to early user feedback.

### Measures and Outcomes

Post-pilot qualitative feedback was obtained from 20 of 32 participants (62.5%) via a structured survey with open-ended questions. The patient survey comprised both closed and open-ended items across four domains: (1) usability and engagement (ease of use, frequency of check-ins, usefulness of reminders, clarity of symptom questions); (2) perceived reassurance and psychological impact (including reassurance, anxiety related to alerts, and perceived safety); (3) impact on care (including symptom self-management, perceived responsiveness to alerts, and overall satisfaction); and (4) acceptability and future use (including recommendation to others and interest in future participation). Free-text responses explored perceived benefits, challenges, and suggested improvements. The complete survey instrument is provided in Multimedia Appendix 2. Survey responses were collected using Google Forms and analyzed descriptively and thematically. Four structured clinician debriefs were conducted at weeks 3, 5, 8, and 10 using a semistructured format focusing on (1) impact on situational awareness and clinical prioritization, (2) workflow integration within existing AO pathways, (3) alert appropriateness and specificity, (4) usability of the dashboard interface, and (5) perceived impact on workload and service delivery. Thematic analysis was conducted using an inductive approach, with themes subsequently mapped to CFIR domains to support the structured interpretation of implementation determinants.

Primary feasibility outcomes were recruitment and retention, engagement (completed check-ins divided by expected patient-days), and alert distribution by acuity tier. Secondary outcomes included the proportion of actionable episodes, escalation pathways (telephone management, OHAU assessment, ED referral, direct admission), timeliness of clinical response for in-hours red alerts (defined as alerts submitted during dashboard-monitored hours, Monday to Friday 9 AM to 5 PM, distinct from the 24/7 AO helpline), out-of-hours escalation behavior, and safety signals (missed deterioration, delayed escalation, inappropriate reassurance). User experience outcomes were captured via patient surveys and clinician structured debriefs.

Potential safety signals were assessed using predefined case-finding and structured review. All participants were cross-referenced against OHAU attendances, ED presentations, and unplanned hospital admissions. For each acute care encounter, case notes were reviewed alongside dashboard audit logs, including symptom check-ins and alerts in the preceding 48 hours. Safety signals were predefined as (1) missed deterioration, (2) delayed escalation attributable to the workflow, (3) inappropriate reassurance leading to delayed help-seeking, or (4) technical failure contributing to harm.

### Data Analysis

All analyses were descriptive (counts, proportions, medians, and ranges). Temporal trends in daily check-in volume were visualized using run charts with median lines. Qualitative feedback was reviewed thematically by 2 team members and reconciled through discussion. Given the quality improvement design and absence of a contemporaneous comparator, inferential statistical analyses were not performed.

### Ethical Considerations

The work was delivered as a Trust-approved quality improvement initiative (reference: QI-AO-2025-03) and did not require research ethics committee review, in line with UK Health Research Authority guidance. A Data Protection Impact Assessment was completed and approved through University Hospitals of Leicester NHS Trust information governance processes (dated July 28, 2025). This manuscript follows SQUIRE (Standards for Quality Improvement Reporting Excellence) 2.0 reporting guidance [10].

## Results

### Baseline AO Service Context

Table 1 summarizes the baseline AO helpline activity. Baseline service data are reported to contextualize operational workload rather than for formal comparison with the pilot period, providing insight into underlying AO demand and the operational context within which digital triage would be deployed. In the 18 months preceding implementation (January 2023 to June 2024), the AO helpline received a median of 107 calls per week (IQR 95-118). Approximately 70% of the calls were from patients receiving active SACT. Call outcomes were as follows: 52% managed with advice without admission, 14% escalated to OHAU review, 24% referred to ED or admitted, and 10% administrative/other.

**Table 1:** Baseline acute oncology helpline activity (January 2023 to June 2024).

Metric		Values
Median (IQR) calls per week		107 (95-118)
**Caller type (%)^a^**		
	Oncology patients receiving active SACT^b^	~70
	Hematology patients receiving active SACT^c^	~30
Managed with telephone advice (%, no admission)^a^		52
Escalated to OHAU (%)^a,d^		14
Referred to ED^e^ or admitted (%)^a^		24
Administrative/other (%)^a^		10

^a^These percentages are derived from aggregate service call logs over an 18-month period. Absolute call counts are not available from the call log system and are therefore not reported.

^b^SACT: systemic anticancer therapy.

^c^Hematology patients included in helpline activity were those receiving active systemic anticancer therapy (eg, chemotherapy for hematological malignancy) and managed within the joint Oncology–Hematology Assessment Unit pathway. This reflects the mixed oncology/hematology case-mix of the Acute Oncology helpline service and does not imply hematology patients were enrolled in the OncsCare pilot evaluation.

^d^OHAU: Oncology–Hematology Assessment Unit.

^e^ED: emergency department.

### Recruitment and Retention

**Figure 1:**
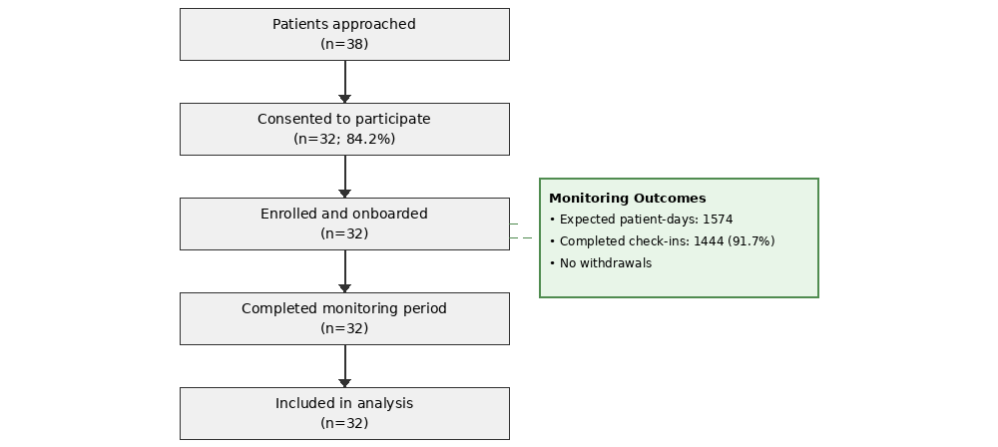
CONSORT (Consolidated Standards of Reporting Trials) flow diagram of participant recruitment, retention, and inclusion in analysis during the 10-week service evaluation. Of the 38 patients assessed for eligibility, 6 were excluded (concern about daily reporting burden n=3, lack of smartphone ownership n=2, anticipated prolonged travel n=1). Thirty-two patients were enrolled; zero withdrew during the 10-week pilot. Overall check-in completion: 91.7% (1444/1574 expected patient-days).

Figure 1 shows the recruitment and retention flowchart. Thirty-eight patients were approached; 32 (84.2%) consented, and none withdrew during the 10-week evaluation. Reasons for declining were concern about daily reporting burden (n=3), lack of smartphone ownership (n=2), and planned travel (n=1).

The full participant demographics and clinical characteristics are presented in Table 2. The median age was 55 (range 33-77) years; 21 (65.6%) participants were females. Tumor groups were predominantly breast (n=18, 56.3%), with representation from colorectal (n=4), lungs (n=3), and other sites. Most participants managed the application independently (n=29, 90.6%); 3 used caregiver support. Treatment intent was curative in 17 (53.1%) participants and palliative in 15 (46.9%) participants. By treatment setting, 9 (28.1%) were receiving neoadjuvant therapy, 6 (18.8%) adjuvant, 2 (6.3%) other curative, and 15 (46.9%) metastatic. Treatment modality comprised chemotherapy alone in 16 (50%), chemo-immunotherapy in 10 (31.3%), chemotherapy with targeted therapy in 1 (3.1%), and targeted or biologic therapy alone in 5 (15.6%). ECOG performance status was 0 in 15 (46.9%), 1 in 16 (50%), and 2 in 1 (3.1%). Participants represented a heterogeneous AO population: 53.1% (n=17) were receiving treatment with curative intent (including neoadjuvant and adjuvant settings) and 46.9% (n=15) palliative, with substantial representation of chemoimmunotherapy regimens (n=10, 31.3%).

**Table 2:** Participant demographics and clinical characteristics (N=32). Treatment modality categories are mutually exclusive as recorded at enrollment.

Characteristic		Values
Age (y), median (range)		55 (33-77)
Female sex, n (%)		21 (65.6)
**Tumor site, n (%)**		
	Breast	18 (56.3)
	Colorectal	4 (12.5)
	Lung	3 (9.4)
	Testicular	2 (6.3)
	Other (gastric, ovarian, esophageal, pancreatic, sarcoma)	5 (15.6)
**Treatment intent, n (%)**		
	Curative	17 (53.1)
	Palliative	15 (46.9)
**Treatment setting, n (%)**		
	Neoadjuvant	9 (28.1)
	Adjuvant	6 (18.8)
	Other curative	2 (6.3)
	Metastatic	15 (46.9)
**Treatment modality, n (%)**		
	Chemotherapy alone	16 (50)
	Chemo-immunotherapy	10 (31.3)
	Chemotherapy + targeted therapy	1 (3.1)
	Targeted/biologic therapy alone	5 (15.6)
**Route of administration, n (%)**		
	Oral SACT^a^	14 (43.8)
	Intravenous SACT	16 (50)
	Subcutaneous immunotherapy	2 (6.3)
**ECOG^b^ performance status, n (%)**		
	ECOG 0	15 (46.9)
	ECOG 1	16 (50)
	ECOG 2	1 (3.1)
Digital self-sufficiency, n (%)		29 (90.6)
Caregiver-assisted, n (%)		3 (9.4)

^a^SACT: systemic anticancer therapy.

^b^ECOG: Eastern Cooperative Oncology Group.

### Objective 1: Engagement and Retention

Across the evaluation, 1444 symptom check-ins were completed (median 44 per participant; range 12-70). Of 1574 expected patient-days, the overall completion rate was 91.7% (1444/1574). Engagement was sustained without marked attrition (Figure S1C in Multimedia Appendix 3). High recruitment rate (32/38, 84.2%) and zero attrition were consistent with sustained engagement throughout the pilot period, supporting the feasibility of this monitoring model in routine outpatient oncology.

### Objective 2: Alert Distribution and Episode-Level Actionability

Across the 10-week evaluation, 1444 check-ins generated 1082 green (74.9%), 228 amber (15.8%), and 134 red (9.3%) alerts. The median number of amber/red alerts per participant was 11 (range 2-31). Red alert distribution was highly skewed: 11 of the participants (34.4%) experienced no red alerts, while the majority of the remainder (14/32, 43.8%) had only 1-3 alerts. A small subset (3/32, 9.4%) accounted for a disproportionate number of alerts, each experiencing ≥10 high-acuity events, suggesting that alert burden is concentrated in a minority of patients. Of 134 red alerts, 69 (51.5%) occurred during staffed monitoring hours and were managed via the dashboard workflow; the remaining 65 (48.5%) occurred out-of-hours and were managed through standard AO helpline and emergency pathways consistent with onboarding safety-netting. Alert activity peaked in mid-August (weeks 5-7), consistent with mid-cycle toxicity periods (Figures S1A-S1C in Multimedia Appendix 3).

**Figure 2:**
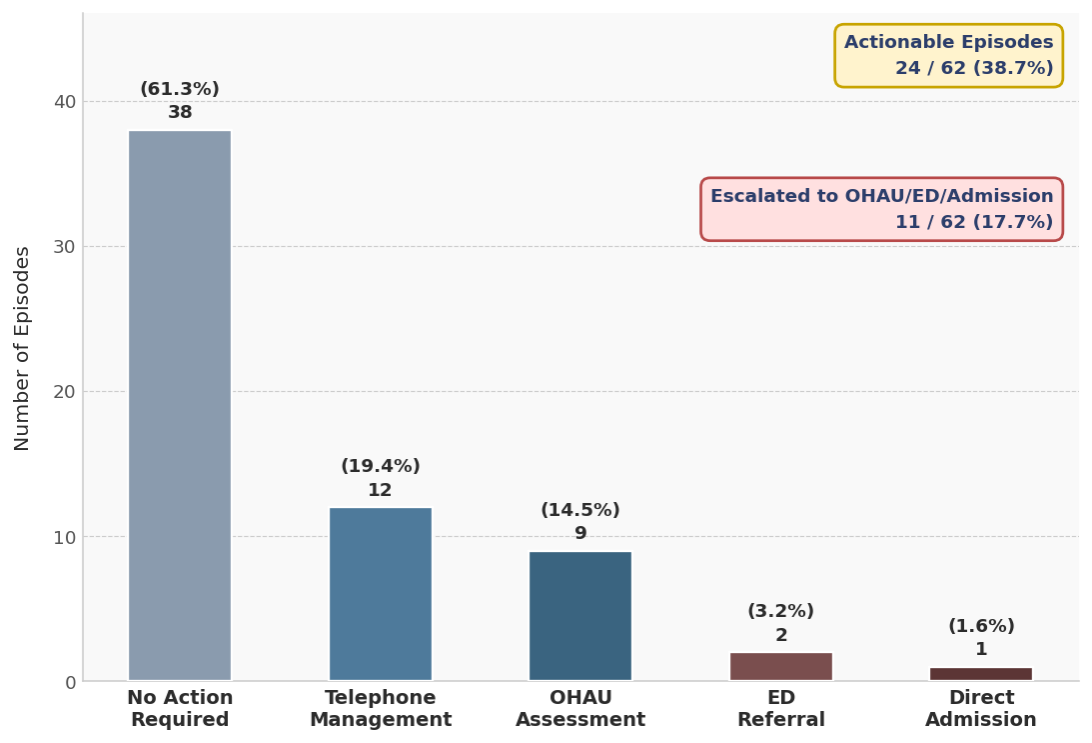
Bar chart showing the distribution of clinical actions across all 62 episodes. Of 62 episodes, 38 (61.3%) required no intervention; among 24 (38.7%) actionable episodes, telephone management (n=12, 50%), OHAU assessment (n=9, 37.5%), ED referral (n=2, 8.3%), and direct admission (n=1, 4.2%) are shown. ED: emergency department; OHAU: Oncology–Hematology Assessment Unit.

**Figure 3:**
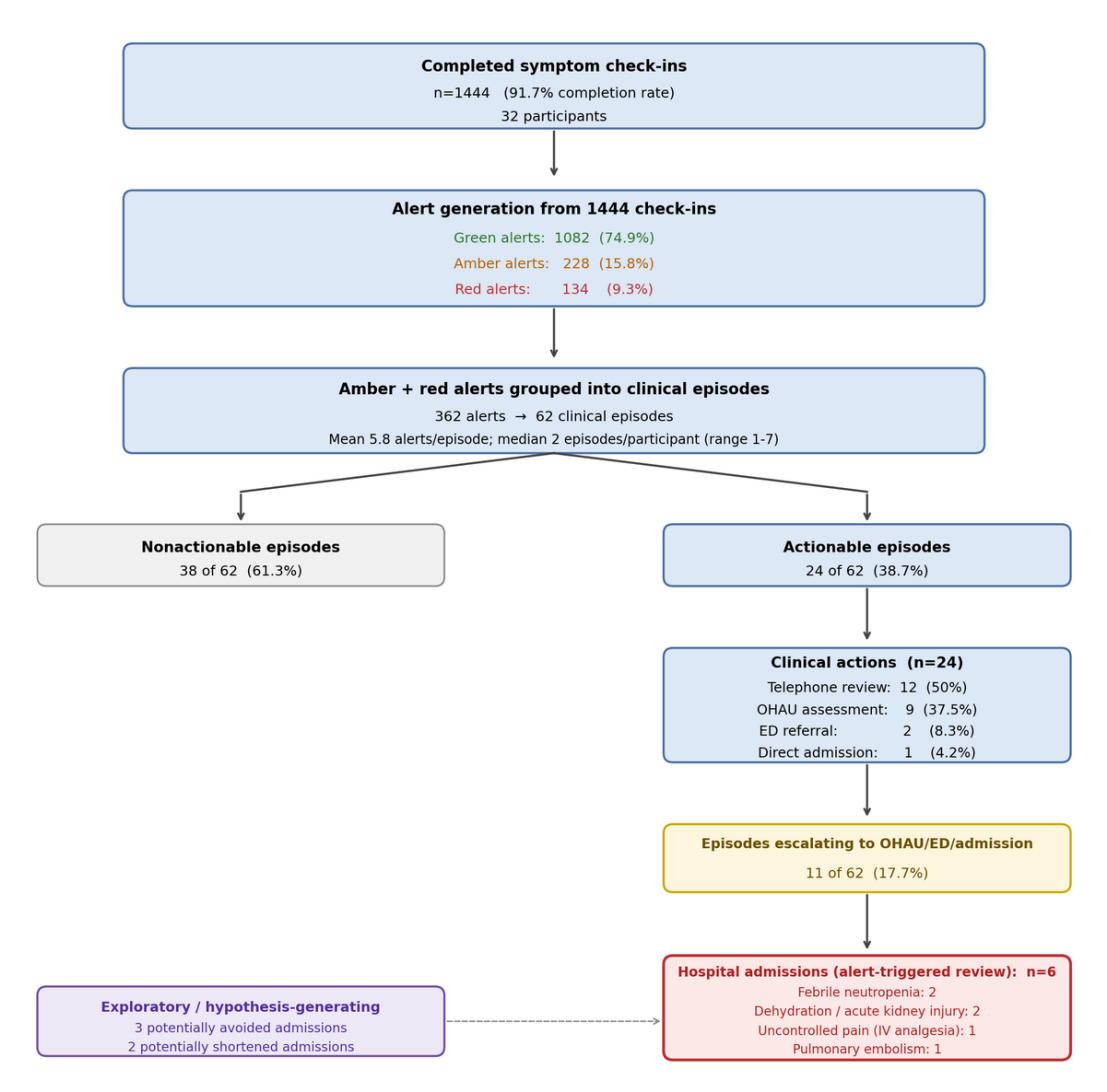
Operational flow from symptom check-ins through episode grouping to clinical outcomes and downstream care pathways. AKI: acute kidney injury; ED: emergency department; FN: febrile neutropenia; IV: intravenous; OHAU: Oncology–Hematology Assessment Unit; PE: pulmonary embolism.

Figure 2 shows the distribution of clinical actions, while Figure 3 illustrates the operational flow from symptom check-ins through episode grouping to clinical outcomes. Table 3 summarizes the episode classifications. Using the prespecified episode grouping approach, 362 amber/red alerts were consolidated into 62 clinical episodes (mean 5.8 alerts per episode; range 1-18)—a 5.8-fold reduction in discrete review events. The median number of episodes per participant was 2 (range 1-7). Twenty-four episodes (38.7%) were clinically actionable; 38 (61.3%) were reviewed without intervention as symptoms were mild, self-limiting, or already under management.

**Table 3:** Clinical episode classification and outcomes (n=62 episodes).

Episode category			Values, n (%)
Total clinical episodes			62 (100)
**Episode actionability**			
	Clinically actionable	24 (38.7)	
	Non-actionable (reviewed, no intervention required)	38 (61.3)	
**Clinical actions (actionable episodes, n=24)^a^**			
	Telephone clinical review with safety-netting advice	12 (50)	
	OHAU^b^ assessment	9 (37.5)	
	ED^c^ referral	2 (8.3)	
	Direct hospital admission	1 (4.2)	
**Escalation outcomes (all episodes, n=62)^d^**			
	Episodes escalating to OHAU/ED/admission	11 (17.7)	
	Episodes managed with telephone advice only	12 (19.4)	
	Episodes reviewed, no intervention required	38 (61.3)	
Hospital admissions following alert-triggered review			6 (9.7)

^a^Percentages for clinical actions are of actionable episodes (n=24).

^b^OHAU: Oncology–Hematology Assessment Unit.

^c^ED: emergency department.

^d^Percentages for escalation outcomes are of all episodes (n=62).

Among the 24 actionable episodes, telephone clinical review with safety-netting advice was the most frequent (n=12, 50%), followed by OHAU assessment (n=9, 37.5%), ED referral (n=2, 8.3%), and direct admission (n=1, 4.2%). Overall, 11 of 62 episodes (17.7%) resulted in escalation to OHAU, ED, or admission. Six hospital admissions occurred following alert-triggered review: febrile neutropenia (n=2), dehydration with acute kidney injury (n=2), uncontrolled pain requiring intravenous analgesia (n=1), and pulmonary embolism (n=1).

As a hypothesis-generating, exploratory analysis only, 5 cases were identified prospectively by the clinical team as potential examples where earlier digital detection may have supported admission avoidance or shortened length of stay. These were subsequently independently adjudicated via structured case-note review: 2 potentially shortened admissions (febrile neutropenia managed with timely antibiotics); 2 potentially avoided admissions (severe diarrhea managed in OHAU with IV fluids); and 1 complex episode managed safely without admission.

### Objectives 3 and 4: Escalation Pathways, Timeliness, and Safety Signals

Using dashboard audit logs and retrospective review of acute care encounters, 2 instances were identified in which participants submitted a green check-in earlier in the day but subsequently presented to hospital later the same day. In both cases, presenting complaints (acute abdominal pain and febrile neutropenia, respectively) developed after the morning check-in; both patients appropriately activated standard urgent care pathways. Neither case represented inappropriate reassurance attributable to the intervention. These cases illustrate a fundamental limitation of point-in-time symptom assessment: a single daily check-in cannot detect acute intraday deterioration, and the platform does not provide continuous monitoring.

No other intervention-related safety signals were identified. All 69 red alerts submitted during in-hours monitoring periods (Monday to Friday 9 AM to 5 PM) were reviewed: 66 (95.7%) of the 69 alerts on the same day. Median time to first clinical response was 47 minutes (range 12 minutes to 6 hours). Three reviews exceeded 2 hours due to concurrent OHAU activity; in each instance, patients were contacted and managed via an established helpline pathway with no patient harm resulting.

### Objective 5: Patient and Clinician Experience

Post-pilot qualitative feedback was obtained from 20 of the 32 participants (62.5%). Four primary themes were identified: reassurance through active monitoring (17/20, 85%), ease of use and low burden (20/20, 100%), clarity of symptom reporting and guidance (15/20, 75%), and confidence in timely clinical response (14/20, 70%). Illustrative quotes are provided below, with additional quotations in Multimedia Appendix 4. Regarding reassurance, one participant described, “Having a daily reminder to check in and feeling I was being supported.” Another noted, “The thought of someone monitoring your symptoms.” Regarding ease of use and low burden, “Easy to use. Clear instructions… and reassuring knowing there was someone checking on any symptoms that were worrying.” Some participants highlighted areas for refinement, including the need for clearer symptom grading and additional contextual input options.

These qualitative findings align with the high engagement and completion rates observed, suggesting that perceived clinical oversight and low interaction burden were key drivers of sustained use. Within the CFIR framework, these themes map primarily to inner setting (workflow compatibility, perceived value) and intervention characteristics (usability, clarity of outputs), supporting the feasibility of the integration into routine AO practice. One clinician highlighted, “Ability to see at a glance the symptoms and concerns of patients and easily identify those needing help.”

Clinician debriefs (n=3 clinicians; 4 sessions at weeks 3, 5, 8, and 10) identified five themes: (1) improved situational awareness across the outpatient cohort (3/3, 100%), (2) real-time alerts with clear audit trail (3/3, 100%), (3) ease of use with minimal onboarding (2/3, 67%), (4) early escalation potentially preventing or shortening admissions (3/3, 100%), and (5) need for alert specificity refinement and contextual data integration (3/3, 100%). All clinicians reported no increase in the perceived workload.

## Discussion

### Principal Findings

In this 10-week service evaluation, implementation of a UKONS-informed digital symptom triage platform within routine NHS AO pathways was feasible and acceptable, with sustained engagement (1444/1574, 91.7% completion), timely clinician response to in-hours high-acuity alerts (median 47 minutes), and no intervention-attributable safety signals identified via structured case-finding. The limited sample size and single-center design constrain the conclusions that can be drawn, and larger multicenter evaluation is required. This evaluation provides an operational signal that structured UKONS-aligned digital triage can be integrated into routine AO workflows without increasing clinician workload.

The findings of this evaluation are consistent with prior trials of ePRO monitoring, which have demonstrated improved symptom control, quality of life, and survival when structured symptom reporting is coupled with clinician response [3,4,7]. However, these systems were primarily designed for longitudinal monitoring and research workflows. In contrast, OncsCare was designed for direct integration into AO triage pathways, mapping symptoms to nationally adopted UKONS escalation thresholds and supporting real-time clinical decision-making. Unlike prior ePRO systems, which rely on the clinician interpretation of longitudinal symptom trends, OncsCare provides structured triage outputs aligned with existing clinical frameworks. This distinction highlights a potential evolution from monitoring-focused platforms to operational, workflow-integrated digital safety-netting interventions.

### Alert Specificity and Algorithm Refinement

Only 38.7% (24/62) of the clinical episodes were actionable, meaning the majority of the episodes reviewed did not require clinical intervention. While this did not produce evidence of alert fatigue in this small cohort, a high nonactionable rate has implications for adoption readiness. Immediate next steps should prioritize practical implementation work: refinement of alert thresholds and composite alert rules informed by adjudicated episode outcomes, integration of treatment schedule data to contextualize expected versus unexpected toxicity, and incorporation of laboratory results (particularly neutrophil counts for temperature alerts). Adoption-readiness testing across two NHS sites, design and specification refinement, and early health economic modelling are the near-term priorities before broader scale-up is considered. The skewed distribution of the red alerts—with a small minority of patients accounting for a disproportionate alert burden—suggests that threshold calibration and targeted monitoring strategies should be priorities for future implementation, potentially enabling more efficient resource allocation.

This proportion of actionable episodes suggests that triage outputs retained clinical specificity while avoiding indiscriminate escalation—a balance that will require ongoing calibration as the platform scales. The patient perspective on nonactionable alerts also warrants attention. This evaluation did not systematically capture how participants perceived episodes in which a red-tier alert was raised but no clinical action was taken. Patients who receive urgent red-tier guidance but are subsequently told no action is needed may experience confusion or eroded trust in the alert system. Future studies should include patient-reported experience of nonactionable alerts as a specific outcome measure. Patient feedback also identified specific usability refinements for future iteration: clearer differentiation of repeat notifications, the addition of free-text fields for contextual symptom detail, and improved anchoring of symptom severity scales. These insights directly informed planned algorithm and interface refinement and are consistent with the iterative PDSA approach adopted throughout the evaluation.

### Safety in Digital Urgent-Care Workflows

Safety in digital urgent-care workflows requires layered safeguards. OncsCare was implemented as an adjunct to existing AO pathways with bounded monitoring hours, explicit in-app and onboarding safety-netting, UKONS-informed escalation thresholds, audit logging, and retrospective safety case-finding linked to objective acute care encounters. The 2 same-day presentations reinforce that the platform complements rather than replaces clinical vigilance and patient self-escalation. Importantly, no cases of harm attributable to delayed or inappropriate triage were identified. Larger multicenter evaluation with prespecified safety end points and independent adjudication is required before safety conclusions can be drawn. The 5 potentially avoided or shortened admissions identified in exploratory analysis should be interpreted cautiously: these classifications are counterfactual and subject to ascertainment bias and are presented solely to inform hypothesis generation for future controlled evaluation.

### Operational Implementation and Practical Implications

This evaluation highlights a transferable design pattern: mapping patient-reported symptoms to established clinical triage frameworks, coupled with episode-level grouping to reflect operational workload rather than raw alert volume. This reflects a shift from reactive, contact-driven triage to proactive, structured situational awareness at the service level. Episode-based grouping represents a further shift from alert-level monitoring to clinically meaningful workload representation, reducing discrete review events by 5.8-fold in this evaluation. Near-term practical implications include augmenting SACT helplines with structured longitudinal symptom visibility, embedding digital monitoring within routine clinical governance using PDSA cycles, and developing audit infrastructure aligned with UKAOS recommendations for standardized data capture. Health economic evaluation is a priority for any future multicenter evaluation. A key perceived benefit, reported by all 3 clinicians in debrief, was enhanced cohort-level oversight, enabling risk-stratified prioritization rather than reliance on episodic, patient-initiated contact. This positions OncsCare within a broader landscape of digital triage approaches. In contrast, reactive models, including patient-initiated helplines and e-consult pathways, do not provide longitudinal visibility between contacts. OncsCare offers a clinically structured intermediate: structured daily reporting mapped to nationally adopted escalation thresholds, with episode-level workload consolidation designed to support sustainable clinical review. Future comparative evaluations should examine whether proactive symptom monitoring confers measurable benefits in safe escalation versus reactive-only alternatives.

### Digital Equity and Accessibility

This evaluation demonstrates high engagement among participants with smartphone access and digital confidence. However, eligibility criteria requiring smartphone ownership and English-language consent likely excluded digitally marginalized populations. Digital health interventions risk widening health inequities if deployment favors populations with existing digital access and literacy [11,12]. Future implementations should adopt deliberate equity-focused strategies, including formal digital literacy assessment [13], alternative access modalities (SMS text message–based reporting, telephone check-ins), multilingual interfaces, and device loan programs. These considerations are addressed further in the Limitations section.

### Implementation Science Framework Mapping

Retrospective CFIR mapping identified key adoption facilitators in the inner setting domain (leadership engagement, workflow compatibility) and outer setting domain (alignment with UKAOS policy framework) [9]. Barriers to wider adoption include intervention characteristics (alert specificity), inner setting (limited electronic health record integration), and equity of access (individual characteristics and outer setting). Within the RE-AIM framework [8], this evaluation addressed Reach (32 patients enrolled; 32/38, 84.2% consent rate), Adoption (clinician engagement with dashboard workflow and participation in structured debriefs), and Implementation fidelity (1444/1574, 91.7% check-in completion; 66/69, 95.7% same-day red alert review). Effectiveness (clinical outcomes) and Maintenance (sustained adoption beyond the pilot) will be formally evaluated in the prospective multicenter study.

### Comparison With Other ePRO Platforms

While eRAPID [7] and PRO-CTCAE [14] have demonstrated efficacy in longitudinal symptom tracking and research contexts, OncsCare was designed specifically for operational integration into acute care triage workflows, with UKONS framework mapping, episode-based alert consolidation, and PDSA-driven implementation via routine clinical governance.

### Limitations

This evaluation is limited by its 10-week duration, single-center design, modest sample size (n=32), and absence of a contemporaneous comparator.

#### Selection Bias and Generalizability

Pragmatic recruitment, smartphone ownership requirements, and English-language consent criteria introduce systematic selection bias. Findings should not be generalized to digitally excluded populations, older adults with lower digital literacy, those with limited English, or patients managed at nontertiary centers.

#### Clinical Lead Involvement

The lead author is the developer of the OncsCare platform, creating potential for confirmation and ascertainment bias in episode classification, actionability judgements, and thematic analysis. Independent adjudication of a random 21% sample (92.3% concordance for episode assignment, 84.6% for actionability) provides partial mitigation. Future evaluations should involve fully independent adjudication and should separate the platform developer from primary data collection roles.

#### Point-In-Time Assessment and Intraday Deterioration

A single daily check-in cannot detect acute clinical deterioration between submissions. The 2 same-day presentations illustrate this directly. The platform does not provide continuous monitoring, and safety-netting messaging must consistently emphasize this constraint.

#### Out-Of-Hours Coverage

Dashboard monitoring was restricted to weekday in-hours periods (Monday to Friday, 9 AM to 5 PM). This is distinct from the AO helpline, which operates 24/7 across two 12-hour nursing shifts and remained fully available throughout the evaluation. Nearly half of the red alerts (65/134, 48.5%) were submitted out-of-hours and were not captured by the dashboard workflow. While safety-netting directed patients to the 24/7 helpline and emergency services, we cannot assess whether digital monitoring influenced out-of-hours escalation behavior.

#### Alert Specificity

Only 38.7% (24/62) of the episodes were actionable. Episode grouping relied on rule-based logic rather than validated clinical algorithms, and threshold calibrations were not validated against an independent dataset.

#### Subtle Harms

Safety case-finding was linked to objective acute care encounters. Subtle harms not resulting in OHAU/ED/admission—such as patient anxiety from nonactionable red alerts or delayed self-escalation—may not have been detected.

#### Digital Equity in Future Implementations

Deliberate equity-focused strategies will be required for wider deployment, including formal digital literacy assessment using validated tools such as eHEALS [13]; alternative access modalities for non–smartphone users; multilingual interfaces co-designed with ethnically diverse patient groups; device loan programs; and explicit subgroup analysis by age, ethnicity, socioeconomic status, and digital literacy in the planned multicenter study (2027-2028).

#### Health Economics

Cost per avoided admission, clinician dashboard review time, and wider health system impact were not formally evaluated and are priorities for the multicenter evaluation.

### Conclusions

This single-center service evaluation demonstrates the feasibility of implementing UKONS-informed digital symptom triage with episode-based review within routine NHS AO workflows. High patient engagement, episode-level actionability, timely clinical response, and absence of intervention-attributable safety signals in this small single-center evaluation support further multicenter evaluation. Planned next steps include adoption-readiness testing across 2 NHS sites, alert algorithm refinement, and early health economic modelling, with a view to informing the design of a comparative multicenter study.

## Appendix

Multimedia Appendix 1 Plan–Do–Study–Act (PDSA) cycles undertaken during the 10-week service evaluation, detailing iterative refinements to the OncsCare platform, including alert logic, workflow integration, and clinician review processes within acute oncology pathways.

Multimedia Appendix 2 Patient feedback survey instrument administered post-pilot to OncsCare participants (20/32, 62.5%). Survey sections cover demographics, app usability, symptom reporting experience, impact on care, and recommendations for future development.

Multimedia Appendix 3 Additional visualizations of alert patterns during the 10-week evaluation period. (A) Distribution of alert acuity over time showing green, amber, and red alerts. (B) Heat map of symptom acuity illustrating temporal patterns across participants. (C) Run chart of red alerts over time showing variation in high-acuity symptom reporting.

Multimedia Appendix 4 Full thematic analysis of patient and clinician feedback from the OncsCare service evaluation. Part A presents four patient themes derived from post-pilot survey free-text responses (n=20). Part B presents five clinician themes derived from structured debrief sessions and post-pilot survey (n=3). Representative quotes and supporting quantitative data are provided for each theme.

Multimedia Appendix 5 SQUIRE 2.0 checklist.

## Abbreviations

AO: Acute Oncology
CFIR: Consolidated Framework for Implementation Research
ED: emergency department
ePRO: electronic patient-reported outcome
OHAU: Oncology–Hematology Assessment Unit
PDSA: Plan–Do–Study–Act
RE-AIM: Reach, Effectiveness, Adoption, Implementation, Maintenance
SACT: systemic anticancer therapy
SQUIRE: Standards for Quality Improvement Reporting Excellence
UKAOS: UK Acute Oncology Society
UKONS: UK Oncology Nursing Society

## Data Availability

Deidentified aggregate data supporting the findings are available from the corresponding author on reasonable request, subject to University Hospitals of Leicester NHS Trust information governance approvals. Individual-level datasets are not publicly available due to privacy and consent constraints.
