# Supplementary material for: Implementing UK Oncology Nursing Society–Informed Digital Symptom Triage With Episode-Based Review in Routine NHS Acute Oncology: Service Evaluation
Source: JMIR Cancer. 2026 May 21;12:e92586. doi: 10.2196/92586 (PMC13237529; doi:10.2196/92586)
Supplement: Multimedia Appendix 1 [file cancer_v12i1e92586_app1.docx]

**PDSA Cycle Details**

This table describes the four Plan-Do-Study-Act (PDSA) cycles implemented during the 10-week evaluation to iteratively refine the OncsCare platform and its integration into acute oncology workflows.

| **PDSA Cycle** | **Timeframe** | **Focus Area** | **Changes Implemented** | **Outcome/Learning** |
| --- | --- | --- | --- | --- |
| 1 | Pre-implementation (4–10 July 2025) | Onboarding workflow + go-live readiness | Brief pre-implementation testing refined onboarding scripts and safety messaging prior to first patient enrolment on 11 July 2025 | First-check-in completion within 24 hours post-registration (once go-live began) |
| 2 | Weeks 2–3 (8–21 July 2025) | Alert threshold calibration; Addressing initial alert volume and appropriateness | Alert threshold refinements (15 July 2025): • Removed false-positive red alerts for low-normal temperature (36.0–37.4°C changed from amber to green) • Introduced composite sepsis risk rule: temperature 35.5–35.9°C escalates to Red only if accompanied by fatigue ≥Grade 2 OR any other symptom ≥Grade 2 • Revised total score thresholds: Green 0–4 (previously 0–2), Amber 5–7 (previously 3–5), Red ≥8 (previously ≥6) • Maintained hard red triggers: fever ≥37.5°C, hypothermia <35.5°C, severe dyspnoea (Grade 3), bleeding ≥Grade 2, any two Grade 3 symptoms • Modified fatigue grading descriptors to better distinguish Grade 1 vs Grade 2 | Amber alert proportion decreased from 72% to 63% of total alerts. Clinical review feedback indicated improved alert relevance and reduced false-positive red alerts for borderline temperature readings |
| 3 | Weeks 3–5 (22 July – 4 Aug 2025) | Dashboard workflow efficiency; Reducing duplicate alert handling and improving clinician review experience | Introduced episode-based grouping to reduce duplicate handling of related alerts. Standardised documentation fields for episode outcomes (reviewed/no action, advice, OHAU/ED/admission) | Clinicians reported improved usability and reduced perceived duplication in handling related alerts following introduction of episode-based review. Feedback informed refinement of episode definitions and dashboard documentation fields |
| 4 | Weeks 6–10 (5 Aug – 19 Sept 2025) | Safety messaging reinforcement; Addressing patient concerns about monitoring hours and emergency pathways | Reinforced onboarding script and written information to emphasise that monitoring is not continuous and to restate use of the AO helpline, ED or emergency services for urgent symptoms. Re-emphasised existing tier-specific post-check-in guidance (green reassurance; amber monitoring; red urgent escalation) during follow-up contacts. Reinforced use of existing reminder notifications and escalation messaging based on early patient feedback regarding out-of-hours concerns | Patient feedback indicated improved understanding of monitoring limitations and escalation routes. Ongoing safety messaging was retained as a core component for future scale-up |

**Key Principles Applied Across PDSA Cycles:**

• Rapid cycle testing: Changes implemented within 3–5 days of identifying issue

• Multi-stakeholder input: Patient feedback collected iteratively during the pilot, clinician debriefs (n=4 clinicians), and project lead review informed each cycle

• Safety-first approach: Any changes affecting alert thresholds or safety messaging were reviewed by clinical lead and AO service manager before implementation

• Documentation: All changes logged in project change register with rationale, approval, and implementation date

*PDSA = Plan-Do-Study-Act quality improvement methodology; OHAU = Oncology–Haematology Assessment Unit; ED = emergency department; AO = acute oncology. Cycles were overlapping rather than strictly sequential, with some concurrent refinements occurring across multiple domains. The composite sepsis risk rule for low-normal temperature was introduced based on early identification of potential sepsis presentations with non-febrile hypothermia combined with other systemic symptoms.*
