# Supplementary material for: Implementing UK Oncology Nursing Society–Informed Digital Symptom Triage With Episode-Based Review in Routine NHS Acute Oncology: Service Evaluation
Source: JMIR Cancer. 2026 May 21;12:e92586. doi: 10.2196/92586 (PMC13237529; doi:10.2196/92586)
Supplement: Multimedia Appendix 2 [file cancer_v12i1e92586_app2.docx]

**OncsCare Service Evaluation — July–September 2025**

University Hospitals of Leicester NHS Trust

# **Supplementary File 3**

**Patient Feedback Survey Instrument**

*Administered post-pilot to participants who completed the 10-week OncsCare service evaluation (n = 20 of 32 respondents, 62.5%). Responses were used to generate qualitative feedback and thematic analysis reported in the main manuscript and Supplementary File 4.*

## **Section 1: About You**

| **Question** | **Response Options** | **n (%)** |
| --- | --- | --- |
| What is your age group? | Under 40 40–49 50–59 60–69 70+ | 2 (10%) 2 (10%) 3 (15%) 6 (30%) 7 (35%) |
| What is your gender? | Female Male Other / prefer to self-describe | 14 (70%) 6 (30%) 0 (0%) |
| Do you identify with an ethnic minority background? | Yes No | 5 (25%) 15 (75%) |
| Were you completing the app check-ins: | Yourself With support from a carer/family member Carer completed on your behalf | 19 (95%) 1 (5%) 0 (0%) |

## **Section 2: Using OncsCare**

| **Question** | **Response Options** | **n (%)** |
| --- | --- | --- |
| How easy was the OncsCare app to use? (1 = Very difficult → 5 = Very easy) | 1 2 3 4 5 | 0 (0%) 0 (0%) 0 (0%) 1 (5%) 19 (95%) |
| How often did you complete your daily check-ins? | Every day Most days Some days Rarely | 14 (70%) 6 (30%) 0 (0%) 0 (0%) |
| Did the reminders (notifications/texts) help you remember to complete check-ins? | Yes, very helpful Somewhat helpful Not helpful I didn't notice them | 16 (80%) 2 (10%) 0 (0%) 2 (10%) |
| How clear were the symptom questions? (1 = Not clear at all → 5 = Very clear) | 1 2 3 4 5 | 0 (0%) 0 (0%) 0 (0%) 6 (30%) 14 (70%) |
| Did you feel the alert messages (green/amber/red) were: | Reassuring and clear Clear but made me anxious Confusing I didn't see any alerts | 17 (85%) 2 (10%) 0 (0%) 1 (5%) |
| Did the alerts ever make you feel more anxious than reassured? | Yes Sometimes No | 1 (5%) 5 (25%) 14 (70%) |
| Was completing the daily check-ins manageable for you? | Yes, always Sometimes No, it felt burdensome | 16 (80%) 4 (20%) 0 (0%) |
| Did you experience any difficulties with the app due to language, literacy, or technology? | Yes No Not sure | 0 (0%) 20 (100%) 0 (0%) |
| Did you feel reassured knowing that nurses were monitoring your submissions? | Yes, definitely Somewhat Not really Not at all | 18 (90%) 2 (10%) 0 (0%) 0 (0%) |

## **Section 3: Impact on Your Care**

| **Question** | **Response Options** | **n (%)** |
| --- | --- | --- |
| Do you think using OncsCare helped you feel safer during your treatment? | Yes Unsure No | 19 (95%) 1 (5%) 0 (0%) |
| Did OncsCare help you manage symptoms at home without going to hospital? | Yes Unsure No | 16 (80%) 2 (10%) 2 (10%) |
| If you had a serious alert, how quickly did you feel your concern was responded to? | Within 2 hours Within 4 hours Same day Longer than a day Not applicable | 4 (20%) 0 (0%) 2 (10%) 0 (0%) 14 (70%) |
| Overall, how satisfied were you with OncsCare during the pilot? (1 = Very dissatisfied → 5 = Very satisfied) | 1 2 3 4 5 | 2 (10%) 0 (0%) 0 (0%) 5 (25%) 13 (65%) |

## **Section 4: Improvements and Next Steps**

*The following questions were free-text open-ended responses.*

**Q1.** What was the best thing about using OncsCare?

**Q2.** What was the most difficult thing about using OncsCare?

**Q3.** What improvements would you like to see in the future?

*Free-text responses to these questions are analysed thematically in Supplementary File 4.*

## **Section 5: Recommendation**

| **Question** | **Response Options** | **n (%)** |
| --- | --- | --- |
| Would you recommend OncsCare to other patients receiving cancer treatment? | Yes, definitely Maybe No | 18 (90%) 2 (10%) 0 (0%) |
| Would you be interested in taking part in future workshops to help shape OncsCare? | Yes Maybe No | 12 (60%) 7 (35%) 1 (5%) |

*Survey administered via Google Forms. Responses are anonymous and were used solely for service evaluation purposes.*
