# Supplementary figures and images for: Implementing UK Oncology Nursing Society–Informed Digital Symptom Triage With Episode-Based Review in Routine NHS Acute Oncology: Service Evaluation
Source: JMIR Cancer. 2026 May 21;12:e92586. doi: 10.2196/92586 (PMC13237529; doi:10.2196/92586)

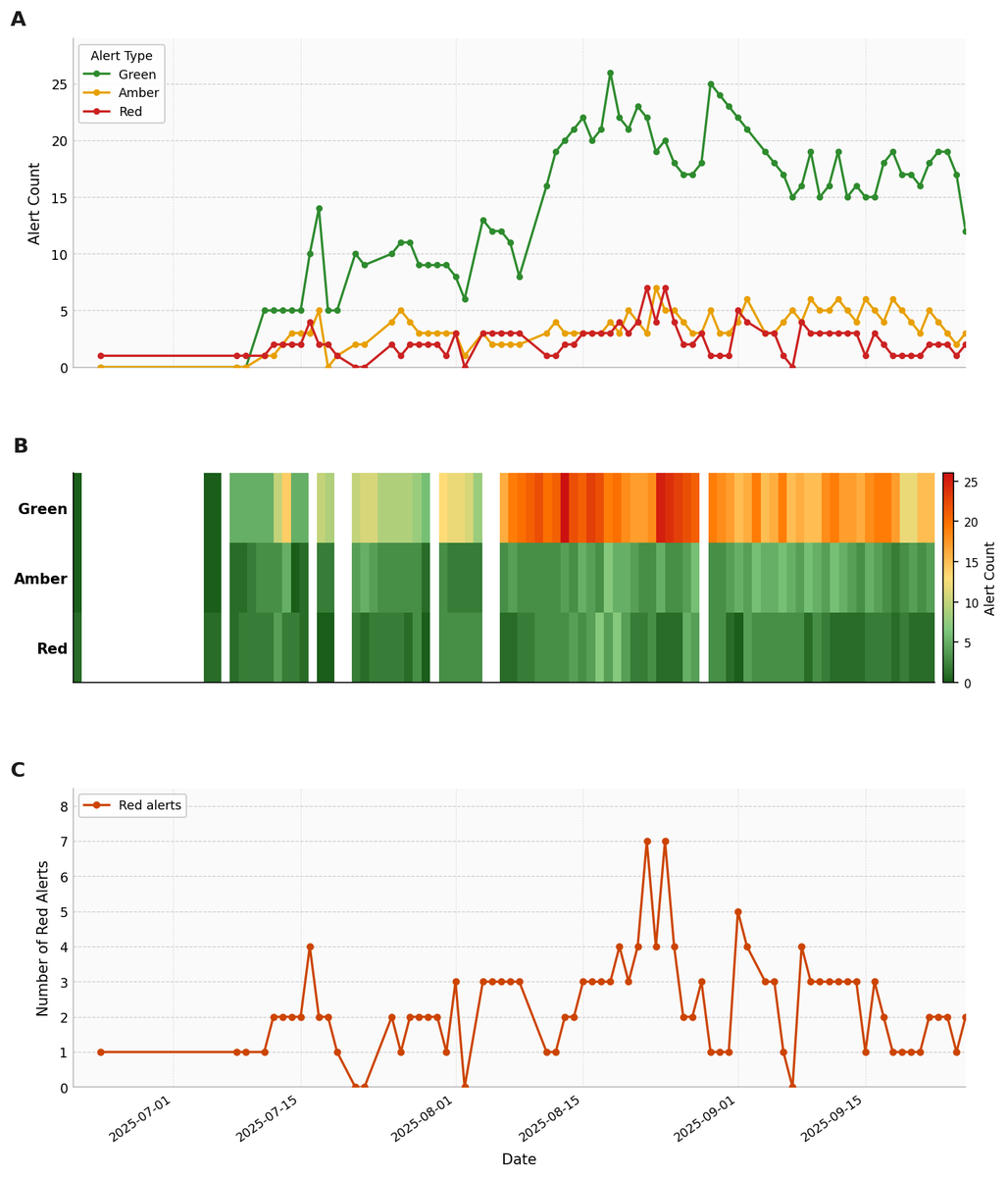

Supplement: Multimedia Appendix 3 [file cancer_v12i1e92586_app3.png]
