# Supplementary material for: Implementing UK Oncology Nursing Society–Informed Digital Symptom Triage With Episode-Based Review in Routine NHS Acute Oncology: Service Evaluation
Source: JMIR Cancer. 2026 May 21;12:e92586. doi: 10.2196/92586 (PMC13237529; doi:10.2196/92586)
Supplement: Multimedia Appendix 4 [file cancer_v12i1e92586_app4.docx]

**OncsCare Service Evaluation — July–September 2025**

University Hospitals of Leicester NHS Trust

# **Supplementary File 4**

**Full Thematic Analysis: Patient and Clinician Feedback**

*Thematic analysis was conducted independently by two team members (an acute oncology nurse and the project lead), with themes reconciled through discussion. Patient free-text responses were obtained from the post-pilot structured survey (n = 20 respondents). Clinician themes were derived from four structured debrief sessions (n = 3 clinicians, weeks 3, 5, 8, and 10) and the post-pilot clinician/AO nurses feedback survey. Participant identifiers are used throughout to protect anonymity (Patient 1–20; Clinician A–C).*

# **Part A: Patient Thematic Analysis**

Twenty of 32 participants (62.5%) completed the post-pilot patient feedback survey. Four primary themes were identified across responses to open-ended questions and supported by quantitative survey items.

**Theme 1: Reassurance Through Active Monitoring** (17/20, 85%)

Patients consistently reported that knowing a clinical team was actively monitoring their daily submissions provided meaningful reassurance during treatment. This theme emerged most strongly in responses to the question ‘What was the best thing about using OncsCare?’ and was supported by 90% of respondents agreeing or strongly agreeing that they felt reassured by nurse monitoring.

**Representative quotes:**

*“Having a nurse monitor you day to day.”* — Patient 3

*“I found the daily contact reassuring.”* — Patient 7

*“Knowing that anything that needed to be checked on was going to be.”* — Patient 11

*“The thought of someone monitoring your symptoms.”* — Patient 14

*“Easy to use. Clear instructions on how to use it and was reassuring knowing there was someone checking on any symptoms that were worrying. They were dealt with quickly.”* — Patient 18

Supporting quantitative data: 18/20 (90%) felt reassured knowing nurses were monitoring; 19/20 (95%) felt safer during treatment; 17/20 (85%) rated alert messages as ‘reassuring and clear’.

**Theme 2: Ease of Use and Low Burden** (20/20, 100%)

All 20 respondents reported the application was easy or very easy to use. The minimal time required for daily check-ins was highlighted as a key facilitator of consistent engagement. Nineteen of 20 respondents (95%) rated usability as 5/5 (very easy). Completion burden was considered manageable by 80% of respondents.

**Representative quotes:**

*“Easy to use.”* — Patient 1

*“Quick and easy.”* — Patient 9

*“User friendly.”* — Patient 16

*“I think that it’s easy to use so doesn’t really need any improvements.”* — Patient 20

*“Easy to use. Control of my symptoms.”* — Patient 6

Supporting quantitative data: 19/20 (95%) rated usability 5/5; 16/20 (80%) reported check-ins were always manageable; 0/20 reported the app felt burdensome; 0/20 reported difficulties due to language, literacy, or technology.

**Theme 3: Clarity of Symptom Reporting and Guidance** (15/20, 75%)

Most patients found the symptom questions clear and the alert tier guidance understandable. However, a minority raised constructive concerns about question specificity, the absence of a free-text field for elaboration, and ambiguity around symptom thresholds. These responses were identified as negative and dissenting feedback and are preserved here to ensure balance and to inform future algorithm refinement.

**Positive responses:**

*“Understanding potential symptoms and their severity classification.”* — Patient 19

*“It said immediately if I needed to take further action.”* — Patient 5

*“Symptoms were similar, common, and recognised by professionals.”* — Patient 8

**Constructive / dissenting feedback:**

*“Some questions too generic/ambiguous and did not allow to elaborate.”* — Patient 12

*“Not having a comment box to write any other concerns. The app was very easy to use so nothing difficult.”* — Patient 15

*“A wider reply choice or an option to write what was actually happening.”* — Patient 17

*“A time frame for the symptoms would make it more clear, e.g. ‘In the last 24 hours have you had…’”* — Patient 19

*“Ability to describe in more detail, for example where pain was or bleeding was.”* — Patient 13

Supporting quantitative data: 14/20 (70%) rated symptom question clarity 5/5 (very clear); 6/20 (30%) rated 4/5; 0/20 rated below 4.

**Theme 4: Confidence in Timely Clinical Response** (14/20, 70%)

Patients who experienced a serious alert reported confidence in the speed of clinical response. Of the 6 patients who reported receiving a response (i.e., excluding ‘not applicable’ responses), 4 (67%) were responded to within 2 hours and 2 (33%) on the same day. This theme also intersects with Theme 1, as the anticipated responsiveness reinforced reassurance even among patients who did not trigger a high-acuity alert.

**Representative quotes:**

*“Having a daily reminder to check in and feeling I was being supported.”* — Patient 2

*“Easy to use. Clear instructions on how to use it and was reassuring knowing there was someone checking on any symptoms that were worrying. They were dealt with quickly.”* — Patient 18

Supporting quantitative data: 4/6 patients with serious alerts (67%) responded to within 2 hours; 2/6 (33%) same day; 0 waited longer than one day; 13/20 (65%) rated overall satisfaction 5/5; median overall satisfaction 5/5.

# **Part B: Clinician Thematic Analysis**

Three clinicians participated in four structured debrief sessions (weeks 3, 5, 8, and 10) and completed the post-pilot clinician/AO nurses feedback survey. Respondents comprised one acute oncology nurse, one oncology registrar, and one oncology consultant (33.3% each). Five themes were identified.

**Theme 1: Improved Situational Awareness Across the Outpatient Cohort** (3/3, 100%)

All three clinicians highlighted the dashboard’s ability to provide a real-time longitudinal view of the outpatient cohort as a meaningful operational benefit not achievable through standard helpline triage.

**Representative quotes:**

*“Dashboard visibility across multiple patients.”* — Clinician A

*“Ability to see at a glance the symptoms and concerns of patients and easily identify those needing help.”* — Clinician C

*“The pilot has demonstrated that OncsCare is feasible, safe, and well-accepted by staff and patients. It provided real-time oversight of patient symptoms and enabled earlier escalation in several cases that likely prevented or shortened admissions.”* — Clinician B

Supporting quantitative data: 2/3 (67%) agreed OncsCare contributed to earlier identification or prevention of deterioration (strongly agree); 1/3 (33%) neutral; 0/3 disagreed. 2/3 (67%) reported OncsCare integrated completely or partly into existing workflow.

**Theme 2: Real-Time Alerts With Clear Audit Trail** (3/3, 100%)

Clinicians valued the timestamped audit trail and real-time alert functionality as tools that supported accountability and retrospective case review. This was noted as a feature not available in existing AO pathways.

**Representative quotes:**

*“Real-time alerts with clear timestamping and audit trail.”* — Clinician A

*“Ability to stratify urgency and prioritise response.”* — Clinician A

*“Excellent integration with multiple teams.”* — Clinician C

Supporting quantitative data: 1/3 (33%) felt alerts reflected clinical urgency very well; 1/3 (33%) reasonably well; 1/3 (33%) neutral. All 3/3 (100%) would recommend continued use of OncsCare if further funding were available.

**Theme 3: Ease of Use With Minimal Onboarding** (2/3, 67%)

Two of three clinicians specifically noted the low onboarding burden and intuitive interface as facilitators of adoption. One clinician (33%) reported the platform was partially but not fully integrated into their existing workflow, suggesting some residual friction.

**Representative quotes:**

*“Ease of use for both nurses and clinicians, minimal onboarding required.”* — Clinician A

*“Good interface.”* — Clinician C

Supporting quantitative data: 3/3 (100%) rated the platform ‘Very easy’ to use; 2/3 (67%) reported ‘Very confident’ using the dashboard; 1/3 (33%) ‘Somewhat confident’; 2/3 (67%) reported OncsCare made urgent oncology calls ‘much easier’ or ‘somewhat easier’ to manage.

**Theme 4: Early Escalation Potentially Preventing or Shortening Admissions** (3/3, 100%)

All three clinicians reported that OncsCare had supported earlier identification of deteriorating patients, with the perception in several cases that intervention had prevented or shortened a hospital admission. These are exploratory, hypothesis-generating observations and are reported as such; they are not supported by formal comparative data.

**Representative quotes:**

*“Early escalation potentially preventing or shortening admissions.”* — Clinician A

*“The pilot has demonstrated that OncsCare is feasible, safe, and well-accepted by staff and patients. It provided real-time oversight of patient symptoms and enabled earlier escalation in several cases that likely prevented or shortened admissions.”* — Clinician B

*Note: Exploratory assessment identified 3 potentially avoided admissions and 2 potentially shortened admissions. These are hypothesis-generating observations only, derived from structured case-note review without a contemporaneous comparator, and should not be interpreted as evidence of causal impact on admission rates.*

Supporting quantitative data: 2/3 (67%) strongly agreed OncsCare contributed to earlier identification or prevention of deterioration; 1/3 (33%) neutral.

**Theme 5: Need for Alert Specificity Refinement and Contextual Data Integration** (3/3, 100%)

All three clinicians raised the need for refinement of alert thresholds, noting that some low-grade alerts (particularly temperature alerts in patients with known menopausal symptoms) were not clinically actionable. Suggestions included contextual notes, EPR integration, and improved UKONS alignment. One clinician also raised a safety concern regarding patient non-adherence to red-tier escalation advice.

**Representative quotes:**

*“No concerns that posed risk. A few alerts were triggered by low-grade, non-actionable fevers (e.g., menopausal symptoms), but these were quickly identified as such. The logic for fever thresholds may benefit from refinement or context adjustment in future builds.”* — Clinician B

*“Fine-tuning symptom thresholds (particularly temperature and fatigue clusters). Option for contextual notes or ‘acknowledged, monitoring’ status on dashboard. Enhanced weekend or out-of-hours alert routing. Integration with electronic patient record (EPR) would make workflow seamless.”* — Clinician A

*“I feel it needs to align better to the UKONS tool — yes it aligns but in some cases if a patient said yes to a question they were automatically triggered as Amber, although in some cases when triaged by a nurse they may well have been a green. There were also concerns as some patients although advised to call the emergency line, did not follow the advice.”* — Clinician C

*The concern raised by Clinician C regarding patient non-adherence to red-tier escalation advice is noted. This was reviewed as part of predefined safety case-finding and no adverse outcomes attributable to non-adherence were identified. This finding informs future onboarding and safety-netting design.*

Supporting quantitative data: 1/3 (33%) reported a safety concern (low-grade, non-actionable temperature alerts); these were promptly identified and did not result in clinical harm. 0/3 reported concerns posing patient risk.

## **Summary of Themes**

| **Theme** | **Description** | **Proportion** | **Key Supporting Evidence** |
| --- | --- | --- | --- |
| **P1** | Reassurance through active monitoring | 17/20 (85%) | 90% felt reassured by nurse monitoring; 95% felt safer during treatment |
| **P2** | Ease of use and low burden | 20/20 (100%) | 95% rated usability 5/5; 0% reported burden |
| **P3** | Clarity of symptom reporting and guidance | 15/20 (75%) | 70% rated clarity 5/5; constructive feedback on ambiguity noted |
| **P4** | Confidence in timely clinical response | 14/20 (70%) | 67% of alerted patients responded within 2 hours; 65% overall satisfaction 5/5 |
| **C1** | Improved situational awareness | 3/3 (100%) | Dashboard visibility; 67% strongly agreed on earlier identification |
| **C2** | Real-time alerts with clear audit trail | 3/3 (100%) | Timestamping and audit trail valued; all 3 recommended continued use |
| **C3** | Ease of use with minimal onboarding | 2/3 (67%) | 100% rated platform very easy; 67% very confident on dashboard |
| **C4** | Early escalation, potentially preventing admissions | 3/3 (100%) | 3 potentially avoided, 2 potentially shortened admissions (exploratory) |
| **C5** | Need for alert specificity refinement | 3/3 (100%) | Temperature/fatigue threshold refinement; EPR integration suggested |

*P = Patient theme; C = Clinician theme. Patient themes derived from post-pilot survey free-text responses (n = 20). Clinician themes derived from structured debrief sessions and post-pilot survey (n = 3).*

*Thematic analysis was performed by two independent team members with reconciliation through discussion, consistent with standard qualitative methods for service evaluation.*
