# Supplementary material for: Implementing UK Oncology Nursing Society–Informed Digital Symptom Triage With Episode-Based Review in Routine NHS Acute Oncology: Service Evaluation
Source: JMIR Cancer. 2026 May 21;12:e92586. doi: 10.2196/92586 (PMC13237529; doi:10.2196/92586)
Supplement: Multimedia Appendix 5 [file cancer_v12i1e92586_app5.pdf]

Supplementary File 2: SQUIRE 2.0 Checklist

Standards for Quality Improvement Reporting Excellence (SQUIRE 2.0)

Manuscript title: Implementing UKONS-Informed Digital Symptom Triage with Episode-Based Review in Routine NHS Acute Oncology: A Service Evaluation

Item	SQUIRE 2.0 Item	Location in Manuscript
**Title and Abstract**		
1	Indicate that the manuscript concerns an initiative to improve healthcare	Title; Keywords; Abstract (structured as quality improvement study)
2	Provide a structured summary of the intervention, methods, results, and conclusions	Abstract (Background, Objective, Methods, Results, Conclusions)
**Introduction**		
3	Problem Description: Nature and significance of the local problem	Introduction, paragraphs 1-2 (reactive helpline model, limited visibility, UKAOS review findings)
4	Available Knowledge: Summary of what is currently known	Introduction, paragraph 3 (ePRO evidence); Discussion: Comparison with other ePRO platforms (Table 4)
5	Rationale: Reasons for undertaking the initiative	Introduction, paragraph 4 (operational gaps, UKAOS recommendations, need for UKONS-informed digital infrastructure)
6	Specific Aims: Purpose of the project and of this report	Introduction, final paragraph (five evaluation objectives)
**Methods**		
7	Context: Contextual elements considered important at outset	Methods: Study design and setting, Baseline service characterisation; Results: Baseline acute oncology service context (Table 1)
8	Intervention: Description of the intervention(s) and their essential functions	Methods: Intervention (OncsCare platform and workflow), Episode grouping approach, PDSA cycles; Figure 1; Supplementary Tables S1-S2
9	Study of the Intervention: Approach chosen for assessing the effectiveness of the intervention	Methods: Outcomes, Safety case-finding and adjudication (predefined), Data analysis
10	Measures: Measures chosen for studying processes and outcomes of the intervention	Methods: Outcomes (engagement, alert distribution, actionability, escalation pathways, timeliness, safety signals, user experience)
11	Analysis: Qualitative and quantitative methods used to draw inferences from the data	Methods: Data analysis (descriptive statistics, thematic analysis, independent adjudication, run charts)
12	Ethical Considerations: Ethical aspects of implementing and studying the intervention	Methods: Ethics, information governance and data protection; Clinical safety and escalation safeguards; Patient and public involvement
**Results**		
13	Results: Initial steps of the intervention and their evolution over time; details of the process measures and outcome	Results: All subsections (Recruitment, Engagement, Alert burden, Episode-level triage, Clinical actions, Safety and timeliness, User experience); Figures 2-7; Tables 1-3
14	Contextual Elements: How contextual elements interacted with the intervention	Methods: Implementation approach and PDSA cycles; Supplementary Table S2; Results: Baseline context, User experience; Discussion: Implementation strategy (CFIR domain mapping)
15	Observed Associations: Associations between outcomes, interventions, and relevant contextual elements	Results: Episode-level triage and clinical actionability, Clinical actions and escalation pathways; Discussion: Operational implementation pattern (episode-based grouping)

16	Unintended Consequences: Observed unexpected benefits, problems, failures, or costs	Results: Safety and timeliness (predefined safety case-finding); Methods: PDSA cycles (threshold refinements, out-of-hours messaging); Supplementary Table S2; Discussion: Limitations (out-of-hours concerns)
17	Missing Data: Completeness of data collection relevant to the original aims	Results: Engagement and completion (91.7% completion rate); Discussion: Limitations (out-of-hours alerts not captured by dashboard, denominators differ from baseline)
**Discussion**		
18	Summary: Key findings, interpretation, and their relevance to the rationale and specific aims	Discussion: Principal findings
19	Interpretation: Nature of the association between the intervention and the outcomes; comparison with findings from other publications	Discussion: Digital equity and accessibility considerations, Safety in digital urgent-care workflows, Operational implementation pattern, Comparison with other ePRO platforms (Table 4), Alignment with UKAOS recommendations
20	Limitations: Limits to the generalisability of the work; factors that might have limited internal validity such as confounding, bias, or imprecision in the design, methods, measurement, or analysis	Discussion: Limitations (single-centre, short duration, no contemporaneous comparator, selection bias, bounded monitoring hours, small sample)
21	Conclusions: Usefulness of the work; sustainability; potential for spread to other contexts; implications for practice and for further study in the field	Discussion: Implementation strategy and theoretical framework (CFIR domain mapping, RE-AIM framework application for multicentre study), Future development; Conclusions (multicentre study planned 2026-2027)
**Other Information**		
22	Funding: Sources of funding that supported this work. Role, if any, of the funding organisation in the design, implementation, interpretation, and reporting	Funding section (internally supported quality improvement initiative, no external funding)

Notes:

- All 22 SQUIRE 2.0 items are addressed comprehensively in the manuscript
- The manuscript follows PDSA methodology with four documented cycles (Supplementary Table S2)
- Safety case-finding was predefined with structured review of all acute care encounters
- Implementation science frameworks (CFIR, RE-AIM) are mapped retrospectively and prospectively
- Digital equity considerations are explicitly addressed in Discussion
- Multicentre evaluation is planned with comparative designs and health economic analysis

Reference:

Ogrinc G, Davies L, Goodman D, Batalden P, Davidoff F, Stevens D. SQUIRE 2.0 (Standards for Quality Improvement Reporting Excellence): revised publication guidelines from a detailed consensus process. BMJ Qual Saf. 2016;25(12):986-992. PMID: 26369893
